# Aqueous humor analyses of diabetic macular edema patients with subretinal fluid

**DOI:** 10.1038/s41598-021-00442-z

**Published:** 2021-10-25

**Authors:** Jin-woo Kwon, Byungjin Kim, Donghyun Jee, Yang kyung Cho

**Affiliations:** Department of Ophthalmology and Visual Science, College of Medicine, St. Vincent’s Hospital, Catholic University of Korea, #93 Jungbu-daero, Paldal-ku, Suwon, 16247 Kyunggi-do Korea

**Keywords:** Cytokines, Diabetes complications, Retinal diseases

## Abstract

We identified treatment-naïve diabetic macular edema (DME) patients with or without subretinal fluid (SRF). We compared their baseline characteristics: aqueous concentrations of interleukin (IL)-1β, IL-2, IL-6, IL-8, IL-10, and IL-17, as well as tumor necrosis factor-α, vascular endothelial growth factor (VEGF), and placental growth factor (PlGF). We also compared fundus and optical coherence tomography (OCT) findings, and responsiveness to anti-VEGF treatments. Of 67 DME patients, 18 (26.87%) had SRF. Compared to the no SRF group, the SRF group had significantly higher levels of IL-6, IL-8, VEGF, and PlGF in aqueous humor. After grouping according to diabetic retinopathy stage, non-proliferative diabetic retinopathy (NPDR) patients with SRF had higher aqueous levels of IL-6 and IL-8, compared to NPDR patients without SRF. Moreover, proliferative diabetic retinopathy (PDR) patients with SRF had higher aqueous levels of VEGF and PlGF, compared to PDR patients without SRF. Fundus and OCT analyses revealed that the SRF group had a greater proportion of patients with succinate or patch-shaped hard exudates involving the macula, and greater central subfield thickness (CST) at baseline. After 6 months of anti-VEGF treatments, the SRF group showed better responsiveness in terms of CST; however, visual acuity was not correlated with responsiveness. Considering higher aqueous levels of VEGFs and pro-inflammatory cytokines, SRF could be a biomarker related to diabetic retinopathy activity. DME patients with SRF showed better anatomical responsiveness to anti-VEGF treatments, but did not show better functional improvement on short-term evaluation compared to those of DME patients without SRF.

## Introduction

Diabetic macular edema (DME) is responsible for visual disturbances in patients with diabetic retinopathy (DR)^1^. A cohort study showed that the DME prevalence was 6.1% in patients with type II diabetes, while a meta-analysis demonstrated that the DME prevalence was 6.81% in these patients^2,3^. Alteration of the blood–retina barrier occurs in DME, which involves multiple cytokines and cells^4,5^.

Development of optical coherence tomography (OCT) techniques allows better visualization of retina, and has enabled early DME detection and treatment^6^. There have been many studies on specific OCT findings (e.g., vitreoretinal abnormality, hyperreflective foci, disorganisation of the retinal inner layers, intraretinal cystoid fluid, and ellipsoid zone [EZ] integrity) for prognosis prediction or selection of proper treatments^7,8^.

A common finding on OCT scans of DME patients is subretinal fluid (SRF); however, the patient prognosis and causative mechanisms have not been fully defined^9^. Some studies have shown that DME patients with SRF exhibit better responsiveness or prognosis in terms of best-corrected visual acuity (BCVA) improvement after anti-vascular endothelial growth factor (VEGF) treatments^10,11^. Conversely, other studies have reported poor prognosis in terms of BCVA after these treatments^12,13^. SRF has reportedly shown better response to intravitreal steroid implants^14^. However, the previous studies included small numbers of patients with heterogenous DR stage or treatment history characteristics^10,12,13^. In addition, few studies have investigated the mechanism of SRF by means of aqueous or vitreous biomarkers^15,16^.

In the present study, we enrolled more patients, and then stratified treatment-naïve DME patients according to the presence of SRF. We compared systemic and ocular factors, including the levels of VEGF and inflammatory cytokines in the aqueous humor. We also identified the prognosis of DME patients with SRF after anti-VEGF treatments.

## Results

We enrolled 67 treatment-naïve centre-involving DME eyes of 67 patients. The mean patient age was 57.66 ± 10.46 years; there were 37 men and 30 women. In terms of DR stages, 22 patients had proliferative DR (PDR; 32.84%) and 45 patients had non-proliferative DR (NPDR; 67.16%). The mean BCVA was 0.56 ± 0.30 logarithm of the minimum angle of resolution (logMAR) and the mean central subfield thickness (CST) was 402.40 ± 99.88 µm at baseline. When classifying the DME morphology as cystoid macular edema (CME) or diffuse retinal thickening (DRT), 35 patients had CME and 32 had DRT. We stratified patients according to their SRF status at baseline; 18 (26.87%) patients had SRF and 49 (73.13%) patients did not. The systemic and ocular characteristics of the patients in each group are summarised in Table 1. CST levels at baseline were higher in the SRF group (*p* = 0.007). In fundus examinations, the SRF group included a greater proportion of patients with succinate or patch-shaped hard exudates (HEs) involving the macula, compared to the non-SRF group (*p* = 0.022). In aqueous cytokine analyses, levels of interleukin (IL)-6, IL-8, VEGF, and placental growth factor (PlGF) were significantly higher in the SRF group than in the non-SRF group (*p* = 0.040, *p* = 0.016, *p* = 0.045, and *p* = 0.015, respectively).

**Table 1:** Demographic and clinical characteristics of DME patients according to SRF status at baseline.

		Without SRF (N = 49)	With SRF (N = 18)	*p*
Systemic factors	Sex (male:female)	26:23	11:7	0.765
	Age (years)	58.00 (53.00; 66.00)	59.00 (47.00; 63.00)	0.449
	Duration of diabetes (years)	6.0 (4.0, 10.0)	9.5 (3.0, 10.0)	0.814
	HbA1C (%)	7.70 (7.10; 8.30)	7.80 (7.30; 8.90)	0.440
	Hypertension	24 (48.98%)	8 (44.44%)	0.957
	Dyslipidaemia	8 (16.33%)	2 (11.11%)	0.717
OCT findings	Baseline CST (µm)	342.0 (328.0, 403.0)	416.5 (364.0, 538.0)	0.007
	CME:DRT	28:21	5:13	0.064
	EZ disruption grade			
	0	27 (55.10%)	9 (50.00%)	0.933
	1	11 (22.45%)	4 (22.22%)	
	2	11 (22.45%)	5 (27.78%)	
Aqueous humor cytokines	IL-1β (pg/mL)	0.00 (0.00, 0.00)	0.00 (0.00, 0.00)	0.897
	IL-2 (pg/mL)	0.00 (0.00, 0.00)	0.00 (0.00, 0.00)	0.735
	IL-6 (pg/mL)	7.46 (3.87, 15.74)	16.33 (6.75, 30.99)	0.040
	IL-8 (pg/mL)	11.83 (8.38, 20.70)	18.30 (14.10, 25.23)	0.016
	IL-10 (pg/mL)	0.65 (0.20, 0.93)	0.79 (0.53, 1.73)	0.290
	IL-17 (pg/mL)	0.68 (0.54, 2.16)	1.24 (0.00, 2.16)	0.824
	TNF-α (pg/mL)	0.00 (0.00, 3.03)	0.28 (0.00, 3.58)	0.556
	VEGF (pg/mL)	66.00 (34.05, 106.84)	100.46 (68.92, 196.12)	0.045
	PlGF (pg/mL)	3.55 (2.41, 5.11)	4.66 (3.74, 15.40)	0.015
Ocular factors	Succinate or patch-shaped HEs involving the macula	11 (22.45%)	10 (55.56%)	0.022
	Baseline BCVA (logMAR)	0.50 (0.20, 0.70)	0.50 (0.40, 1.00)	0.485
	DR stage			
	NPDR	34 (69.39%)	11 (61.11%)	0.729
	PDR	15 (30.61%)	7 (38.89%)	

Values are expressed as interquartile ranges.

*DME* diabetic macular edema, *SRF* subretinal fluid, *HbA1c* glycated haemoglobin, *CST* central subfield thickness, *CME* cystoid macular edema, *DRT* diffuse retinal thickening, *EZ* ellipsoid zone, *IL* interleukin, *TNF* tumour necrosis factor, *VEGF* vascular endothelial growth factor, *PlGF* placental growth factor, *HE* hard exudates, *BCVA* best-corrected visual acuity, *DR* diabetic retinopathy, *NPDR* non-proliferative diabetic retinopathy, *PDR* proliferative diabetic retinopathy.

Classifying DME patients according to DR stage, aqueous levels of IL-6, IL-8, VEGF, and PlGF were also significantly higher in the PDR group than in the NPDR group (*p* = 0.001, *p* = 0.012, *p* = 0.022, and *p* < 0.001, respectively) (Table 2). When subgrouped them according to SRF status, aqueous levels of IL-6 and IL-8 were higher in the NPDR with SRF group than in the NPDR without SRF group (*p* = 0.017 and *p* = 0.005). Moreover, aqueous levels of VEGF and PlGF were higher in the PDR with SRF group than in the PDR without SRF group (*p* = 0.024 and *p* = 0.047) (Table 3).

**Table 2:** Demographic features of aqueous humor depending on DMR staging.

	NPDR (N = 45)	PDR (N = 22)	*p* value
IL-1 (pg/mL)	0.00 (0.00; 0.00)	0.00 (0.00; 0.17)	0.392
IL-2 (pg/mL)	0.00 (0.00; 0.00)	0.00 (0.00; 2.62)	0.392
IL-6 (pg/mL)	5.12 (3.87; 10.51)	17.36 (9.11; 36.02)	0.001
IL-8 (pg/mL)	11.92 (8.70; 17.80)	21.42 (11.90; 30.46)	0.012
IL-10 (pg/mL)	0.65 (0.20; 1.14)	0.74 (0.11; 1.34)	0.763
IL-17 (pg/mL)	0.54 (0.00; 2.16)	1.36 (0.54; 2.55)	0.111
TNF-α (pg/mL)	0.05 (0.00; 3.03)	0.00 (0.00; 3.58)	0.574
VEGF (pg/mL)	68.92 (30.72; 102.10)	107.73 (53.93; 268.88)	0.022
PlGF (pg/mL)	3.55 (2.41; 4.40)	5.91 (3.85; 14.98)	< 0.001

Values are expressed as interquartile ranges.

*IL* interleukin, *TNF* tumor necrosis factor, *VEGF* vascular endothelial growth factor, *PlGF* placental growth factor.

**Table 3:** Demographic and clinical characteristics of DME patients grouped according to DR stage and SRF at baseline.

	DR Stage	NPDR (N = 45)			PDR (N = 22)		
		No SRF (N = 34)	SRF (N = 11)	*p*	No SRF (N = 15)	SRF (N = 7)	*p*
Systemic factors	Sex (male:female)	18:16	5:6	0.932	8:7	6:1	0.193
	Age (years)	60.50 (53.00; 66.00)	61.00 (50.50; 62.50)	0.500	55.00 (48.50; 63.00)	57.00 (45.50; 62.00)	0.972
	Duration of diabetes (years)	7.5 (4.0, 15.0)	10.0 (3.0, 11.5)	0.832	5.0 (2.0, 6.5)	9.0 (5.5, 10.0)	0.285
	HbA1c (%)	7.65 (7.10; 8.30)	8.10 (7.75; 8.95)	0.093	7.80 (7.20; 8.40)	7.30 (6.70; 8.15)	0.502
	Hypertension	20 (58.82%)	3 (27.27%)	0.141	4 (26.67%)	5 (71.43%)	0.074
	Dyslipidaemia	6 (17.65%)	1 (9.09%)	0.663	2 (13.33%)	1 (14.29%)	1.000
OCT findings	Baseline CST (µm)	337.0 (323.0, 423.0)	408.0 (375.0, 547.5)	0.023	361.0 (341.0, 396.0)	422.0 (379.0, 526.0)	0.148
	CME:DRT	20:14	3:8	0.141	7:8	5:2	0.381
	EZ disruption grade						
	0	20 (58.82%)	4 (36.36%)	0.391	7 (46.67%)	5 (71.43%)	0.581
	1	7 (20.59%)	3 (27.27%)		4 (26.67%)	1 (14.29%)	
	2	7 (20.59%)	4 (36.36%)		4 (26.67%)	1 (14.29%)	
Aqueous humor cytokines	IL-1β (pg/mL)	0.00 (0.00, 0.00)	0.00 (0.00, 0.09)	0.331	0.00 (0.00, 0.17)	0.00 (0.00, 0.00)	0.322
	IL-2 (pg/mL)	0.00 (0.00, 0.00)	0.00 (0.00, 0.00)	0.463	0.00 (0.00, 1.31)	0.00 (0.00, 1.81)	0.857
	IL-6 (pg/mL)	4.96 (3.42, 9.28)	16.34 (6.96, 31.43)	0.017	18.40 (9.82, 37.95)	16.32 (9.21, 19.54)	0.581
	IL-8 (pg/mL)	10.88 (7.73, 16.05)	17.80 (13.82, 23.56)	0.005	22.15 (11.02, 36.11)	18.80 (15.14, 26.39)	1.000
	IL-10 (pg/mL)	0.53 (0.20, 0.99)	0.93 (0.53, 1.38)	0.243	0.80 (0.18, 0.89)	0.53 (0.27, 2.15)	1.000
	IL-17 (pg/mL)	0.61 (0.00, 2.16)	0.54 (0.00, 1.76)	0.892	1.36 (0.54, 2.35)	1.36 (0.83, 4.28)	0.640
	TNF-α (pg/mL)	0.05 (0.00, 3.03)	0.51 (0.00, 3.69)	0.438	0.00 (0.00, 2.74)	0.00 (0.00, 2.95)	0.906
	VEGF (pg/mL)	62.35 (30.72, 102.10)	73.52 (38.83, 95.63)	0.833	67.61 (46.20, 168.97)	196.12 (184.46, 428.37)	0.024
	PlGF (pg/mL)	3.01 (1.86, 4.08)	4.13 (2.98, 4.66)	0.143	5.11 (3.17, 11.81)	15.40 (6.79, 18.82)	0.047
Ocular factors	Succinate or patch-shaped HEs involving the macula	9 (26.47%)	5 (45.45%)	0.277	2 (13.33%)	5 (71.43%)	0.014
	Baseline BCVA (logMAR)	0.50 (0.20, 0.70)	0.50 (0.35, 1.00)	0.696	0.70 (0.30, 0.70)	0.50 (0.45, 1.00)	0.587

Values are expressed as interquartile ranges.

*DME* diabetic macular edema, *DR* diabetic retinopathy, *SRF* subretinal fluid, *HbA1c* glycated haemoglobin, *CST* central subfield thickness, *CME* cystoid macular edema, *DRT* diffuse retinal thickening, *EZ* ellipsoid zone, *IL* interleukin, *TNF* tumour necrosis factor, *VEGF* vascular endothelial growth factor, *PlGF* placental growth factor, *HE* hard exudates, *BCVA* best-corrected visual acuity.

During follow-up period, the required number of intravitreal bevacizumab (IVB) treatments averaged 5.01 ± 0.83 per patient, and there was no significant difference between the SRF group and the non-SRF group (5.06 ± 0.80 vs. 5.00 ± 0.84, *p* = 0.809). After treatments with IVB for 6 months, the level of CST reduction was significantly greater in the SRF group than in the non-SRF group (*p* < 0.001). The mean CST tended to be thinner in the SRF group than in the non-SRF group with statistical significance (282.44 ± 82.08 μm vs. 320.86 μm ± 56.06 μm, *p* = 0.048). A significantly greater proportion of patients in the SRF group showed responsiveness to IVBs during the follow-up period (*p* = 0.035). Despite better responsiveness, the SRF group tended to have worse BCVA, compared to the non-SRF group, but this difference was not statistically significant (0.56 ± 0.35 vs. 0.44 ± 0.29, *p* = 0.245) (Table 4).

**Table 4:** Clinical results after anti-VEGF treatments for 6 months in DME patients.

	Without SRF (N = 49)	With SRF (N = 18)	*p*
Baseline CST (µm)	342.0 (328.0, 403.0)	416.5 (364.0, 538.0)	0.007
CST after IVBs (µm)	306.00 (290.00; 350.00)	267.50 (244.00; 348.00)	0.048
CST reduction after IVBs (µm)	42.00 [29.00; 58.00]	174.50 [80.00; 236.00]	< 0.001
BCVA (logMAR) at baseline	0.50 (0.20, 0.70)	0.50 (0.40, 1.00)	0.485
BCVA (logMAR) after IVBs	0.40 (0.20, 0.70)	0.45 (0.30, 1.00)	0.245
BCVA (logMAR) improvement	0.00 (− 0.20; 0.00)	0.00 (− 0.10; 0.00)	0.402
Required number of IVBs	5.00 (4.00; 6.00)	5.00 (4.00; 6.00)	0.816
^†^Poor responsiveness to IVBs	24 (48.98%)	3 (16.67%)	0.035

Values are expressed as interquartile ranges.

*DME* diabetic macular edema, *SRF* subretinal fluid, *CST* central subfield thickness, *BCVA* best-corrected visual acuity, *IVB* intravitreal bevacizumab.

^†^CST ≥ 300 µm or CST reduction < 50 µm after treatment.

## Discussion

We designed this study to identify the association of SRF with aqueous VEGF or inflammatory cytokines, and the association of SRF presence with anti-VEGF responsiveness corresponding to the aqueous biomarkers in DME patients. We found that DME patients with SRF had higher aqueous levels of some pro-inflammatory cytokines and VEGFs. The SRF group included a greater proportion of patients who had succinate or patch-shaped HEs in the fundus examination, compared to the non-SRF group. Analyses of responsiveness showed that patients in the SRF group had better responsiveness to anti-VEGF agents in terms of CST; BCVA improvement was not correlated with responsiveness.

Intravitreal injections of anti-VEGF agents or steroid implants have become the main treatment approach for DME patients^17,18^. Studies using imaging or aqueous humor biomarkers to identify underlying mechanisms and select effective treatments are currently underway^19–21^. Some studies have reported associations between SRF and VEGF or inflammation using aqueous or vitreous sample and associations between SRF and responsiveness to anti-VEGF or steroid implant treatments in DME patients^12,14,15,22^. However, the results have been inconsistent among studies. Here, we improved the study design by including more patients and using stricter criteria, as well as stratification according to DR stage. We assumed that PDR patients might exhibit higher levels of inflammatory cytokines and VEGF. The aqueous levels of IL-6, IL-8, VEGF, and PlGF were higher in the PDR group. We also found that these pro-inflammatory cytokines and VEGFs were elevated in DME patients with SRF. In subgroup analyses according to DR stage, aqueous levels of IL-6 and IL-8 were significantly higher in the NPDR subgroup, while the aqueous level of VEGFs were significantly higher in the PDR subgroup. These findings suggest that SRF could be caused by various mechanisms according to DR stage. Although there were differences in the involved cytokines, the observation that each cytokine was significantly elevated in the SRF subgroup indicates that SRF could be a biomarker for increased DR activity.

We found that large HEs involving the macula were more common in DME patients with SRF. HEs in DME involve leakage of lipoprotein accumulated in the outer plexiform layer, which leads to neurological and photoreceptor damage^23,24^. Although some studies have shown HE reductions after repeated anti-VEGF or steroid intravitreal injection treatments^25,26^, HE depositions within the macula are presumed to indicate worse visual prognosis^27,28^. In our study, despite responsiveness in the SRF group, BCVA improvement was less robust in the SRF group than in the non-SRF group. A factor contributing to this difference may have been the greater proportion of SRF patients with HEs involving the macula at baseline. In addition, two patients with SRF had severe visual impairment due to macular atrophy after HE migration into the subretinal space during anti-VEGF treatments.

In terms of CST responsiveness, the SRF group had better clinical results in our study. Some studies regarding anti-VEGF agents have shown that DME patients with SRF exhibit good responsiveness and better visual prognosis^11,29^. However, another study demonstrated that DME patients with SRF exhibited greater CST, external limiting membrane disruption, and significant macular functional impairment, relative to DME patients without SRF^30^. OCT analyses of the SRF group in this study showed that greater CST at baseline could be attributed to SRF. Because SRF disappeared after anti-VEGF treatments in all the patients during treatments, responsiveness was markedly better in the SRF group. However, in contrast to the previous study, we could not identify significant differences in EZ disruption in the SRF group.

The origin of SRF in DME patients is controversial. Some studies have suggested that SRF originates from retinal pigment epithelium barrier dysfunction induced by chronic oxidative and metabolic stresses^31^. This dysfunction is associated with the release of pro-inflammatory molecules, VEGF-mediated enhanced permeability, matrix metalloprotease-induced proteolysis, and cytoskeletal regulatory proteins^32^. Other studies have suggested that the origin of SRF is related to change in the external limiting membrane barrier^32^. We found that the presence of SRF in patients with DME is associated with both inflammation and VEGF. In addition, because some patients showed HE migration to the subretinal space, there could be connections between the outer retina and subretinal space.

Our study had some limitations. First, the observation period was short. A 2-year study of ranibizumab treatment showed that HEs were reduced after treatment; moreover, the presence of HEs was not a prognostic indicator of poor visual outcomes^25^. Second, although we used strict criteria to exclude eyes with conditions that could affect the aqueous humor cytokines or VEGF level, they could be affected by other conditions including size of new vessel and extent of capillary non-perfusion^33,34^. Third, bevacizumab is known to show less responsiveness compared to that of ranibizumab or aflibercept^35^. Fourth, as SRF per se increases CST and easily disappeared after anti-VEGF treatments, responsiveness could be better in the SRF group.

In summary, SRF in DME patients is presumably associated with both inflammation and VEGF. SRF in DME patients showed better responsiveness to anti-VEGF treatments, but their BCVA outcomes were not correlated with responsiveness.

## Methods

The study protocol adhered to the tenets of the Declaration of Helsinki and was approved by the Institutional Review Board of the Catholic University of Korea. All participants provided written informed consent for the use of their clinical records.

We enrolled treatment-naïve centre-involving DME eyes with CST ≥ 300 µm. Only one eye was enrolled randomly if both eyes met the inclusion criteria. The criteria for exclusion included macular edema due to other causes, as well as any history of uveitis, intraocular surgery including cataract surgery, and/or laser treatments. We measured glycated haemoglobin levels and performed ophthalmic examinations in all patients; these examinations included measurements of BCVA and fundus assessment. CST was automatically measured using Cirrus High-Definition OCT (Carl Zeiss Meditec, Dublin, CA, USA; software version 10.0). EZ disruptions were manually measured within 1000 µm using a horizontal scan centred on the fovea^36^. The image of OCT scans was checked and assessed by two retinal specialists. We classified DME patients as either IVB responsive or poorly responsive. Responsiveness was defined as either CST < 300 µm or a CST reduction of ≥ 50 µm after 6 months of treatment with IVB^37^. IVB treatments were conducted using pro re nata regimen when patients showed CST ≥ 300 µm after three consecutive monthly loading injections. We evaluated BCVA and CST after 6 months of treatment.

### Assessments of cytokines and growth factors

We collected aqueous fluid specimens before first IVB injection, and measured the concentrations of IL-1β, IL-2, IL-6, IL-8, IL-10, and IL-17, as well as PlGF and VEGF, in 75 µL samples of aqueous humor. The corresponding antibodies were immobilised on beads and 75 μL aliquots of Calibrator Diluent RD6–52 (R&D Systems, Minneapolis, MN, USA) were added to the samples. Then the samples were incubated for 2 h after adding beads, for 1 h after adding detection antibodies, and for 30 min after adding streptavidin–phycoerythrin reagent. Samples were analysed using the Luminex xMAP system (Luminex, Austin, TX, USA). All values below the lower detection limit indicated by the manufacturer were considered zero values.

### Statistical evaluation

Statistical analyses were performed using SPSS Statistics for Windows, version 21.0 (SPSS, Chicago, IL, USA). The Mann–Whitney U test, and chi-square test were used to compare values or proportions of patient subgroups. The level of statistical significance was set at *p* < 0.05.

## Data Availability

The datasets generated during and/or analyzed during the current study are available from the corresponding author on reasonable request.
