# Author Correction: Aqueous humor analyses of diabetic macular edema patients with subretinal fluid

**DOI:** 10.1038/s41598-022-05477-4

**Published:** 2022-01-18

**Authors:** Jin‑woo Kwon, Byungjin Kim, Donghyun Jee, Yang kyung Cho

**Affiliations:** Department of Ophthalmology and Visual Science, College of Medicine, St. Vincent’s Hospital, Catholic University of Korea, #93 Jungbu‑daero, Paldal‑ku, Suwon, 16247 Kyunggi‑do Korea

Correction to: *Scientific Reports* 10.1038/s41598-021-00442-z, published online 25 October 2021

The original version of this Article contained an error in the Acknowledgments section.

“This work has supported by the National Research Foundation of Korea (NRF) grant funded by the Korea government (MSIT) (No. 52020A015400343).”

now reads:

“This work has supported by the National Research Foundation of Korea (NRF) grant funded by the Korea government (MSIT) (No. 2020R1G1A1007646).”

The original Article has been corrected.

